# Indigenous Knowledge and Science Unite to Reveal Spatial and Temporal Dimensions of Distributional Shift in Wildlife of Conservation Concern

**DOI:** 10.1371/journal.pone.0101595

**Published:** 2014-07-23

**Authors:** Christina N. Service, Megan S. Adams, Kyle A. Artelle, Paul Paquet, Laura V. Grant, Chris T. Darimont

**Affiliations:** 1 Department of Geography, University of Victoria, Victoria, BC, Canada; 2 Raincoast Conservation Foundation, Denny Island, BC, Canada; 3 Spirit Bear Research Foundation, Klemtu, BC, Canada; 4 Hakai Beach Institute, Heriot Bay, BC, Canada; 5 Earth to Ocean Research Group, Department of Biological Sciences, Simon Fraser University, Burnaby, BC, Canada; Université de Sherbrooke, Canada

## Abstract

Range shifts among wildlife can occur rapidly and impose cascading ecological, economic, and cultural consequences. However, occurrence data used to define distributional limits derived from scientific approaches are often outdated for wide ranging and elusive species, especially in remote environments. Accordingly, our aim was to amalgamate indigenous and western scientific evidence of grizzly bear (*Ursus arctos horribilis*) records and detail a potential range shift on the central coast of British Columbia, Canada. In addition, we test the hypothesis that data from each method yield similar results, as well as illustrate the complementary nature of this coupled approach. Combining information from traditional and local ecological knowledge (TEK/LEK) interviews with remote camera, genetic, and hunting data revealed that grizzly bears are now present on 10 islands outside their current management boundary. LEK interview data suggested this expansion has accelerated over the last 10 years. Both approaches provided complementary details and primarily affirmed one another: all islands with scientific evidence for occupation had consistent TEK/LEK evidence. Moreover, our complementary methods approach enabled a more spatially and temporally detailed account than either method would have afforded alone. In many cases, knowledge already held by local indigenous people could provide timely and inexpensive data about changing ecological processes. However, verifying the accuracy of scientific and experiential knowledge by pairing sources at the same spatial scale allows for increased confidence and detail. A similarly coupled approach may be useful across taxa in many regions.

## Introduction

Distributions of organisms are shaped and re-shaped over geological and ecological timescales. Broadly structured by a suite of natural processes and their interactions, changes to species distributions (hereafter ‘range shifts’), can be driven by abiotic factors (*e.g.*, CO_2_ enrichment, nitrogen deposition, climate; [1]), biotic processes (*e.g*., competition and facilitation [2]), and dispersal capability [3], [4]. In recent history, distributional shifts have often been rapid and associated with human-caused drivers. Causes include climate change, habitat modification, over-exploitation, persecution, introduction of exotic species, and re-introduction of native species (*e.g.*, [5]–[10]). The increased pace of range shifts caused by humans can impose ecological effects on other species, communities, and ecosystems by exposing recipients to novel predation pressure, competition, and diseases [11]. For example, the rapid, human-aided range expansion of the brown tree snake (*Boiga irregularis*) to Guam decimated native bird populations, which in turn reduced the reproductive success of vertebrate-pollinated native plant species [12].

Given such potential impacts, current and accurate knowledge of species distributions comprises a fundamental and important dimension in conservation management. For example, many policy-relevant processes, such as protected areas design, mapping of critical habitat, and land-use planning require distributional data [13], [14]. Moreover, current distribution information can also inform proactive conservation intervention in the face of climate change and other stressors (*e.g.*, [10], [15]). More generally, detecting shifts in species' distributions can signal underlying ecological changes within an ecosystem, providing managers with early insight that changes might be occurring in other species and communities.

Identifying contemporary ecological change requires knowledge of the past. A complementary methods approach that combines traditional and local ecological knowledge with conventional scientific methods can provide data that not only offer detailed occurrence data across large areas but also over long time periods [16]. Traditional ecological knowledge (TEK) of indigenous people is transmitted through generations and revolves around a cumulative body of knowledge, practice, and belief surrounding the relationships of living and nonliving beings with their environment and one another [17]. Local ecological knowledge (LEK), often but not exclusively associated with indigenous people, also provides information about ecosystem change, but is gained from observations over lifetimes and not via inter-generational transmission [18]. However, in practice the distinction between TEK and LEK is often imprecise as they may share many similarities [18], [19]. In contrast, wildlife science uses a variety of empirical techniques that span differing temporal and spatial resolutions and can provide detailed and quantitative information on populations and individuals [18]. Such data, however, are often very expensive to acquire and temporally and spatially limited. Accordingly TEK/LEK data – potentially spanning decades or longer – can be summarized and analyzed to yield information on elusive species across large areas that are otherwise too expensive or difficult to monitor with conventional scientific tools (*e.g.*, [20], [21]). Employing TEK/LEK and wildlife science approaches together might yield more comprehensive and detailed information about changes over time and space than either method alone [22], [23]. Importantly, incorporating TEK/LEK into ecological research also can facilitate the engagement of communities [24], [25]. Social research components of LEK and TEK necessarily include local people and affirms the importance of their contributions [[24]]; in turn, a collaborative methods approach can be a critical first step in establishing more collaborative management [17], [19], [25].

**Figure 1:**
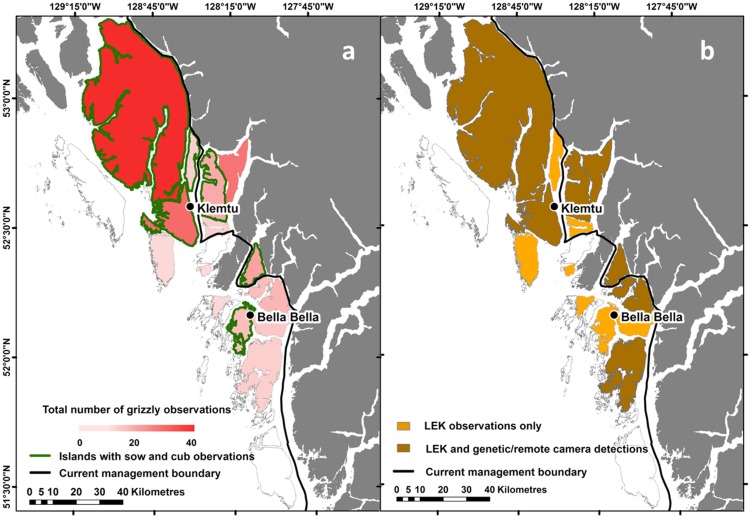
Spatial pattern of evidence of island occupancy and detection type. a) Weight of evidence of grizzly bear (*Ursus arctos horribilis*) occupancy at the island scale within Heiltsuk and Kitasoo/Xai'xais Territories in coastal British Columbia, Canada. Each data type (local ecological knowledge observation, mortality record, genetic ID and remote camera observation) was weighted equally to provide an indication of occupancy rather than bear density. Dark grey areas were not included in our study. The ‘current management boundary' is the westward (i.e., seaward extent) of the Province of British Columbia's Grizzly Bear Population Units, the spatial scale at which grizzly bears are managed in the province. Spatial pattern in data types used to detect grizzly bears (*Ursus arctos horribilis*) in sampled areas within Heiltsuk and Kitasoo/Xai'xais Territories. Eighteen islands were included in the study.

Grizzly bear (*Ursus arctos horribilis*) distribution on the remote and now sparsely populated central coast of British Columbia (BC), Canada, provides an ideal system to examine the temporal and spatial components of potential range shifts using a complementary methods approach. The provincial government's current management boundary, delineated as the western (*i.e.*, seaward) extent of the so-called Grizzly Bear Population Units (GBPUs), is the spatial scale at which grizzly bears are managed for hunting, habitat protection, and human-wildlife conflict [26]. Whereas formalized species accounts do not include details of coastal grizzly distribution at an appropriate spatial scale for this study [27], the current management boundary suggests grizzlies are functionally absent from all but five of the dozens of islands in the vast archipelago ([26], [28]–[32]; T. Hamilton, BC Ministry of Environment, pers. comm; Figure 1). In contrast, local people in the area, including several First Nations communities that still rely heavily on subsistence activities and travel across their expansive Territories, now commonly observe grizzlies on islands. If present, subsistence hunters and fishers are likely to sight and remember large-bodied, diurnal and iconic wildlife, like grizzly bears.

Our overarching aim was to combine TEK, LEK, and western scientific methods (human-caused mortality records, non-invasive genetic sampling, and remote camera data) to record grizzlies on islands and detail a potential range shift. Understanding the potential colonization and occupancy of grizzly bears onto islands in the region has significant conservation implications because these animals possess tremendous ecological, cultural, and economic importance in this area and indeed, where they still exist throughout their global range (*e.g.*, [33]–[36]). More broadly applicable to other areas and taxa, we also test the hypothesis that data from each method yield similar spatial and temporal patterns. Finally, we explore how these approaches reveal complementary spatial and temporal dimensions of data and emerge with broadly applicable conclusions relevant to many systems.

## Methods

### Study area

**Figure 2:**
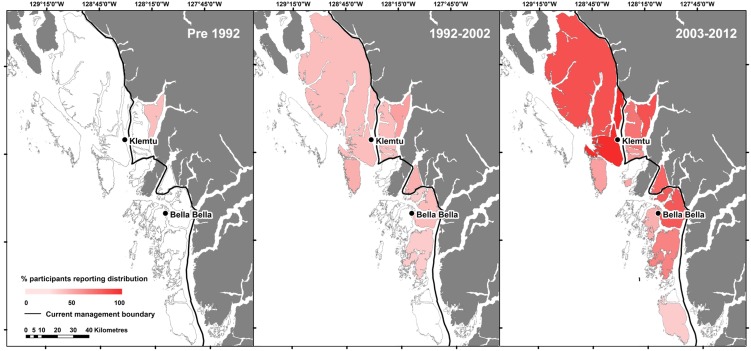
Time series of island grizzly bear occupation over three time periods. Local residents' perception of grizzly bear (*Ursus arctos horribilis*) distribution throughout Heiltsuk and Kitasoo/Xai'xais Territories in coastal British Columbia, Canada during the Pre 1992 (traditional ecological knowledge data), 1992–2002 (local ecological knowledge data) and 2003–2012 (local ecological knowledge data) time periods. Data are reported at the island scale (n = 18 islands) as the percentage of participants who indicated the island was within their area of observation and supported grizzly bears. Dark grey areas were not included in our study and the ‘current management boundary' is the westward (i.e., seaward extent) of the Province of British Columbia's Grizzly Bear Population Units, the spatial scale at which grizzly bears are managed in the province.

The islands and nearby mainland of the central coast of BC (8800 km^2^) occur within a nearly road-less and now sparsely populated region extending from its southern boundary of Calvert Island (51.58° N, 127.81° W) north to Princess Royal Island (53.21° N, 128.05° W) (Figures 1a & 1b). A complex matrix of landmasses, the central coast as a whole is composed of mainland valleys divided by extensive fjords and various sized islands (<1 km^2^ to >2200 km^2^) separated by tidal waters [37]. Eighteen major islands (*i.e*., greater than 45 km^2^) were included in the study area. The closest major island to the mainland (Yeo) is separated by 230 m of tidal water. The Coastal Western Hemlock biogeoclimatic zone dominates low elevations of the region [38]. Potential foods for bears are well distributed across the coastal mainland and islands and include spring (sedges and forbs), summer (berries), and fall (spawning salmonids) resources [39], [40]. Since colonization by Europeans, most people in the area now live in the communities of Bella Bella (Heiltsuk Nation, population ∼2200) and Klemtu (Kitasoo/Xai'xais Nation, population ∼400). Our study area comprised four islands (of five) now recognized by the current management limit and 14 islands beyond this management range (Figures 1 & 2; Table 1).

**Table 1:** Detections of island grizzly bears (*Ursus arctos horribilis*) by data type (presented as raw observations and as detection-per-unit-effort (DPUE) values) within Heiltsuk and Kitasoo/Xai'xais Territories in coastal British Columbia, Canada (n = 18 islands).

Island	Total Remote Camera Observations	Remote Camera Detections Per Trap Night^a^	Total Hair Snag Observations	Hair Snag Year	Hair Snag Detections Per Trap Night^b^^,^ d	LEK Observations	Total Sow/Cub Observations	Mortality Observation
**Aristazabal**	-	-	-	-	-	0	0	0
**Calvert**	-	-	-	-	-	0	0	0
**Campbell**	-	-	0	-	-	6	0	0
**Chatfield**	-	-	2	2009–2012	0.03	6	0	0
**Cunningham**	-	-	1	2009–2012	0.01	7	3	0
**Denny**	-	-	0	2009–2012	0	5	0	0
**Dufferin**	-	-	-	-	-	1	0	0
**Hecate**	-	-	-	-	-	0	0	0
**Hunter**	-	-	-	-	-	1	0	1
**Yeo** **	-	-	1	2009–2012	0.04	12	4	0
**Lady Douglas**	-	-	-	-	-	1	0	0
**Pooley** **	7	0.02	0	2012	0.01	15	0	0
**Price**	0	0	0	2012	0	1	0	0
**Princess Royal**	19	0.03	3	2012	0.02	16	17	1
**Roderick** **	1	0.01	0	2012	0.01	12	3	1
**Sarah**	-	-	-	-	-	9	3	0
**Susan** **	-	-	-	-	-	2	0	0
**Swindle**	1	0.03	1	2012	0.03	22	15	0

a For islands with remote cameras.

b For islands with hair snags.

c Indicates number of observations, not number of individual grizzly bears, across study period.

d Summed across seasons and years.

** Indicates islands within the current management boundary'.

### Data types

#### Interview data

Ethics Statement: The interview component of this study was approved by the Human Research Ethics Board at the University of Victoria (Victoria, BC, Canada - Protocol # 12-385), Heiltsuk Integrated Resource Management Department, and Kitasoo/Xai'xais Integrated Resource Authority. All participants provided written informed consent. Field sampling was approved by the Heiltsuk Integrated Resource Management Department, Kitasoo/Xai'xais Integrated Resource Authority, and BC Parks.

We conducted 22 LEK and seven TEK interviews using a “snowball sampling” method [41]. For TEK interviewees, the Kitasoo/Xai'xais Stewardship Department and the Heiltsuk Integrated Resource Management Department recommended initial participants, who hold traditional oral and observational history knowledge, and who in turn suggested additional experts to interview (University of Victoria Human Research Ethics Approval # 12-385) [22], [42]. The LEK participant pool included mostly indigenous (19 of 22) candidates, including subsistence and commercial fishers, hunters, eco-tour operators, salmon counters, bear viewing guides, and biologists.

Using adapted general guidelines of TEK/LEK data collection, we guided participants through target questions while also recording additional comments [22]. To assess historical grizzly distributions, comprising occupancy over the past century and beyond, we asked TEK participants about their experience and cultural transmission of knowledge about bears. We asked them to depict on their Traditional Territory map where, based on their Nation's oral histories, they would historically expect to see grizzly bears (hereafter ‘Pre 1992’). Given inter-generational transmission of knowledge, we estimate that this TEK ‘temporal window’ provides information about island occupancy that spans centuries or more.

We used a mapping approach to complement interview data. Specifically, LEK participants indicated on a map the islands on which they had observed a grizzly bear. For each observation, participants provided supporting information including the island name, year, and season. We also asked LEK participants to draw on a map the areas they would define as coastal grizzly bear distribution during two timespans (1992–2002, 2003–2012). In addition, an estimate of survey effort for each LEK participant was quantified spatially – by indicating the extent of their area of expertise on a map – and temporally – as the estimated number of years and days per year the participant was in the field and potentially able to observe bears. All TEK/LEK data were verified with interview participants through follow up workshops. Once verified, interview data in transcript form were returned to both the participants and the Nations' Resource Stewardship offices in digital and print versions.

#### Genetic, remote camera, and mortality data

We used genetic data, remote camera images, and mortality records to identify location, date, and – in some cases sex, individual identity, and age class – of grizzly bears detected in the same area from 2009–2012. We sampled genetic data from non-invasive hair-snagging stations baited with a non-reward bait [43], [44]. These stations (n = 33) were part of longitudinal carnivore monitoring programs across 10 islands in Kitasoo/Xai'xais (2012) and Heiltsuk (2009–2012) Territories (Table 1 & 2). In addition, 18 remote trail cameras (n = 1268 trap nights) were deployed on four islands in 2012 (Table 1 & 2). Finally, we queried island locations within the BC Ministry of Environment's kill records from their Compulsory Inspection Database [39], [40], yielding dates, locations, and sexes of known human-killed grizzly bears. All island mortality records (n = 3) were hunted individuals rather than animal control (i.e., human-wildlife conflict) kills (Table 1 & 2).

**Table 2:** Sampling effort of island grizzly bears (*Ursus arctos horribilis*) by data type within Heiltsuk and Kitasoo/Xai'xais Territories in coastal British Columbia, Canada (n = 18 islands).

Island	Number of Remote Cameras	Total Remote Camera Nights^s^	Number of Hair Snag Sites	Hair Snag Year	Total Hair Snag Nights	Number of LEK Interviewees Who Reported on Island^b^	Average Number of Report Days Per Year^c^
**Aristazabal**	0	-	0	-	-	13	120
**Calvert**	0	-	0	-	-	9	120
**Campbell**	0	-	0	-	-	14	142
**Chatfield**	0	-	2	2009–2012	159	14	142
**Cunningham**	0	-	4	2009–2012	304	14	142
**Denny**	0	-	2	2009–2012	178	13	148
**Dufferin**	0	-	0	-	-	13	148
**Hecate**	0	-	0	-	-	9	120
**Hunter**	0	-	0	-	-	12	146
**Yeo** **	0	-	3	2009–2012	221	15	145
**Lady Douglas**	0	-	0	-	-	14	137
**Pooley** **	3	357	3	2012	416	10	136
**Price**	1	23	1	2012	23	13	140
**Princess Royal**	9	661	9	2012	1217	8	140
**Roderick** **	4	192	3	2012	398	11	139
**Sarah**	0	-	0	-	-	8	128
**Susan** **	0	-	0	-	-	14	145
**Swindle**	1	135	1	2012	121	11	140

a For islands with remote cameras.

b Across entire study period.

c Average number of ‘report days’ (i.e., days interviewees reported on for each island) per year from 1992–2012. Data derived from all participants who included.

the island in their area of expertise.

** Indicates islands within the “current management boundary”.

### Analyses

#### Occupancy

We summed the number of grizzly bear observations from all data sources on each island to assess the weight of evidence for contemporary occupancy. We accounted for observations of (genetically) undocumented bears from sightings and camera images equally with known, specific individuals from mortality and genetic data. Accordingly, it is possible that multiple observations were from the same individual bear. However, we did not expect bias across candidate islands in the spatial pattern where such multiple counting might have occurred. We calculated detection-per-unit-effort (DPUE) for all data types except mortality data (Table 1 and Table 2). For remote camera and genetic data, we quantified DPUE for each island by dividing the number of observations by the cumulative number of days each camera (n = 18) or hair snag (n = 33) was employed (Table 2). We standardized LEK observations by dividing the total number of observations for each island by the total number of survey days each interviewee estimated s/he spent within their identified geographic area of expertise. Both survey effort and total number of observations were summed for all participants at the island scale. As level of experience could not be reliably estimated, we assumed the same observational abilities of all participants. To assess which islands contain reproducing females, we used both remote camera and LEK observations to identify all detections of sows with cubs.

#### Temporal Trends in Occupancy

We assessed temporal occupancy across three intervals: Pre 1992 (n = 7 interviewees), 1992–2002 (n = 27 interviewees), and 2003–2012 (n = 27 interviewees). We estimated ‘Pre 1992’ occupancy from TEK data, and 1992–2002 and 2003–2012 periods from LEK data. Because LEK observations are likely increasingly comprehensive with increasing proximity to communities of residence [18], for each island we only used interview data from the closest community. We indexed the total number of observations across all islands by dividing them by the total number of survey days in each year. Survey effort was calculated annually using Equation 1: 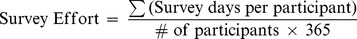
(1)where # of participants is the number of LEK interviewees, 365 denotes the total possible number of survey days a year, and survey days per person indicates the total number of survey days for each participant. The number of island LEK observations per year was then divided by annual survey effort across all islands. This metric allows survey effort to be calculated per annum across all participants for all islands.

## Results

We identified 149 grizzly bear observations across 15 major islands, including 10 islands outside the current grizzly bear management boundary (Figure 1a, Table 1). LEK yielded the largest number of data points relative to other sources, with 110 observations across 15 major islands (Table 1 & 2). Twelve islands hosted more than one observation (mean  = 9.93, range  = 1 to 39) (Table 1; Figure 1a). Across all LEK participants, 86% (19 of 22) reported at least one observation of an island grizzly (Table 1). Data on known individuals (genetic data and mortality records) indicated the presence of at least nine unique individuals (three female, five male and one of unknown gender) across 10 locations on seven islands. Remote cameras yielded a total of 28 observations of grizzlies on four islands (Table 1), two of which are outside of the current management boundary. Sow and cubs were detected in 23 LEK observations and seven remote camera observations across six islands (Table 1).

TEK/LEK data sources revealed a similar pattern of island occupation as did evidence from genetics, cameras, and hunting records; all newly occupied islands identified by scientific evidence were affirmed by complementary TEK/LEK observations (Figure 1b; Table 1). Of the seven islands solely associated with TEK/LEK evidence, all but one (Denny Island) lacked scientific evidence (Figure 1b).

**Figure 3:**
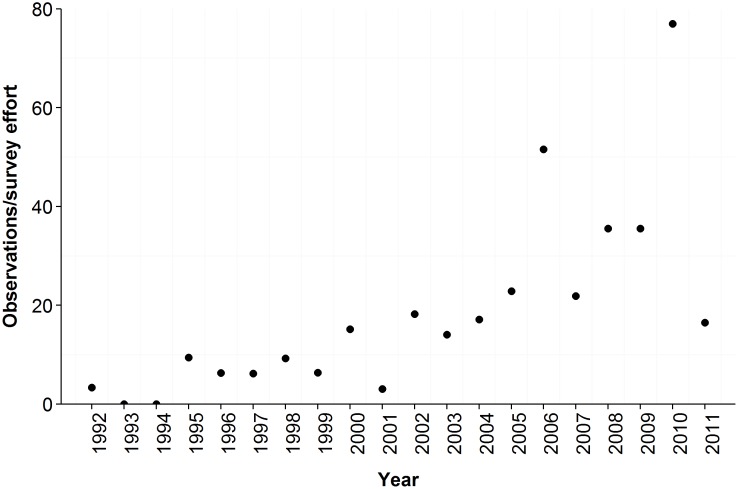
Trend of island grizzly occupation over time as derived from LEK data. Yearly number of island grizzly bear (*Ursus arctos horribilis*) observations per unit survey effort (a summation of all participants' observations across all islands; local ecological knowledge data only) between 1992–2011 in the Traditional Territories of the Heiltsuk and Kitasoo/Xai'xais First Nations in British Columbia, Canada. These observations encompass islands within and outside the current management boundary (n = 18 islands).

Indigenous knowledge additionally yielded historical information suggesting that bear colonization of islands occurred recently, and that the process has increased in pace. Only one of seven TEK interviewees identified grizzly bears on an island before 1992. Accounts of island occurrence, within and outside the currently recognized distribution, were increasingly common and spatially widespread in 1992–2002 and 2003–2012 periods (encompassing 12 and 15 islands, respectively; Figure 2). The total island observations per year, indexed by survey effort, increased between 1992–2011 (Figure 3).

## Discussion

Our approach coupling indigenous knowledge and western science offered multiple lines of evidence that grizzly bears of both sexes have recently colonized 10 islands outside of the currently accepted distribution. Moreover, camera and interview data provided strong evidence for reproductive individuals and their offspring on four islands. Collectively, this suggests a distributional process beyond any exploratory extra-range movements of mobile males. Such an abrupt distributional shift of an apex predator may invoke cascading ecological consequences (*e.g*., [45]). More broadly, the drivers of such change may also act on other less iconic and recognizable species and processes at differing spatial and temporal scales [46], [47].

Though trends in these data are apparent, our approach also presents some inherent limitations. Specifically, although highly useful, TEK/LEK data are not systematic in coverage [23]. Accordingly, use of islands by interviewees likely varies in their mode of use (hunting, fishing, ecotourism, etc.) and as a result the habitat types people frequent may differ. As such, some interviewees may be more likely to encounter grizzly bears than others. Additionally, it is possible that black bears may have been mistaken for grizzly bears by some interviewees. Furthermore, TEK/LEK may not always be appropriately responsive to detect re-colonization and abandonment events that could accompany dynamic distributional processes. Snowball sampling methods may have also excluded some knowledgeable participants who were outside of the social networks of interviewees [48]. Finally, we note that recent observations might be more likely to be remembered and reported. More likely, however, we postulate that older observations of grizzlies outside their known range would have been interpreted as surprising and, accordingly, equally or even more memorable. Despite these limitations, the inclusion of TEK/LEK data provides important process values through the engagement of local people and as a result this approach may facilitate collaborative rather than antagonistic conservation efforts (*e.g.*, [49]–[51]).

Conventional scientific tools, such as remote cameras and genetic identification through hair snagging, also present limitations. Although more systematic in their deployment, the temporal resolution of these data types are limited, both on an annual basis (*i.e.*, spring sampling) and over longer timeframes (most ecological monitoring programs span years rather than decades or centuries) [20]. Additionally, these tools are spatially static and can be impacted by poor placement. Indeed, in the nearly road-less landscape we study, most snag station are set up close to shorelines where they can be accessed by boat. Importantly, though all data sources have potential weaknesses, uniting these independent sources provides increased temporal and spatial detail of the range shift we describe.

Interviewee comments and relevant literature allow us to offer working hypotheses about potential drivers of this shift. Specifically, modifications to the abundance and distribution of food resources as well as changes to intra- and inter- specific competition might be relevant. Many interviewees cited the reduction of salmon (*Oncorhynchus* spp.), which has declined throughout coastal BC, especially since 2000 [52]. As a critical resource that influences ursid body condition, reproductive output, and population dynamics [36], [53], salmon abundance could influence bear distribution; individuals within other carnivore species have expanded or abandoned their range following declines in prey and subsequent increased intraspecific competition (*e.g.*, [54]–[56]). Increasing berry abundance and accessibility, the result of recent logging on islands, might cause individuals on exploratory forays to islands to remain. Grizzly bears in other areas select recently logged habitat to exploit diverse food resources offered in early regeneration stages of disturbed habitat [57]. Alternatively, black bears (*Ursus americanus*) are thought to limit grizzlies via exploitative competition on coastal islands where food resources are more dispersed and more difficult to defend. Acting alone or synergistically with human-caused mortality from trophy hunting of grizzlies, such competition between species has been proposed as a mechanism for excluding grizzly bears from islands [31] or reducing their densities elsewhere [58]. Recent reductions of grizzly bear trophy hunting on the mainland (BC Ministry of Environment, unpublished data), and/or changes in competition from black bears, might have reduced the demographic constraints on grizzly bears, thereby allowing dispersal to nearby islands. Whatever the cause(s) in our system, range shifts observed in closely monitored species may signal changes to underlying ecological conditions and provide resource managers the opportunity to consider and plan for wider implications.

The ecological, cultural, and economic consequences of this distribution shift in coastal grizzly populations might be considerable. As a larger species, grizzly bears outcompete co-occurring black bears (*U. americanus*) for salmon through interference competition [59] and also kill juvenile and adult black bears [60]. In addition, ‘spirit bears’ (*Ursus americanus kermodei*), a rare and geographically restricted white morph of black bears that occur with relatively high frequency on several islands in the area [61], [62], are not only revered culturally but are also economically prized as the foundation of wildlife eco-tourism. Consequently, any mortality or increased avoidance behaviour due to new grizzly bear presence might impact cultural and economic values. Finally, forest management plans for grizzlies – a ‘species of conservation concern’ in BC [63] – carries legally-binding measures to protect high quality habitat wherever grizzly bears occur [30]. Such multifaceted implications would also be likely for range shifts in other high-profile fauna. Indeed, reintroductions of wolves (*Canis lupus*) to the Yellowstone Ecosystem provide a flagship example of the tremendous ecological, cultural, and economic ramifications associated with a change in distribution [64], [65]. Similarly, expansions to sea otter (*Enhydra lutris*) ranges, a system well-suited to TEK/LEK study, carry comparably broad implications for people and ecosystems [66].

Beyond its regional relevance, this study illustrates the synergistic benefits of combining science with TEK/LEK over concurrent and complementary spatial and temporal scales. In our study, both data types predominately affirmed one another; islands without scientific evidence for occupation also lacked concurrent TEK/LEK observations. The co-affirmation of data sources in locations where they overlapped added confidence in patterns observed by LEK where they did not overlap [16]. Genetic and camera data provided precise information on individuals captured, including individual identities, gender, age class, location, and time of visit. These data were captured in all weather and at all hours each day. Costs of these scientific methods, however, necessarily limit their spatial and temporal coverage. Moreover, they can only be employed to capture data in real time. In contrast, although yielding less detailed information on individual bears, TEK/LEK data afforded broader information across a larger area and longer timespan. Finally, despite the inherent value of TEK/LEK, it remains largely ignored as a source of ecological data [19].This case study provides one of the few examples we detected in the literature that illustrates the value in uniting TEK/LEK with scientific methods to provide meaningful input into wildlife management [18], [24], [25], [67].

With careful consideration a TEK/LEK method alone could perform well in other wildlife systems. Such an approach, however, requires careful assessment. Specifically, LEK information might be most useful for questions of distribution but may lack the detail required for other population parameters, such as absolute abundance, without calibration from scientific sources [18], [68]. The case for a TEK/LEK approach alone might be particularly compelling with conspicuous and culturally important focal species in data-deficient regions for which species distributions impose serious management implications and funds for science are scarce. Moreover, as many indigenous governments and societies across the globe play increasingly prominent roles in resource management again [69]–[71], methods that emphasize an integration of, or focus on, existing local knowledge might emerge as default approaches. Such a transition might help overcome the dual common barriers of lack of conservation action due to inadequate data and data deficiency resulting from scarcity of funds. In this way existing indigenous knowledge can be proactively incorporated into management. Such consideration may support a transition to implement management action more rapidly, the timescale in which conservation action is often required.

## Supporting Information

Document S1 **Interview instruments used in Local and Traditional Ecological Knowledge surveys of island grizzly bears (** ***Ursus arctos horribilis*** **) within Heiltsuk and Kitasoo/Xai'xais Territories in coastal British Columbia, Canada.**
(DOCX)
