# Supplementary material for: Indigenous Knowledge and Science Unite to Reveal Spatial and Temporal Dimensions of Distributional Shift in Wildlife of Conservation Concern
Source: PLoS One. 2014 Jul 23;9(7):e101595. doi: 10.1371/journal.pone.0101595 (PMC4108310; doi:10.1371/journal.pone.0101595)
Supplement: Document S1 — Interview instruments used in Local and Traditional Ecological Knowledge surveys of island grizzly bears ( Ursus arctos horribilis ) within Heiltsuk and Kitasoo/Xai'xais Territories in coastal British Columbia, Canada. (DOCX) [file pone.0101595.s001.docx]

## Grizzly Bear Distribution Project Survey

**Name:**

**Participant number (2 digits):**

**Community:**

**Category:**

**Interviewer initials:**

**1. CONTEXTUAL QUESTIONS:**

1. Do you, and/or your Nation, think of mainland and island areas as very different in terms of what animals are present in each area?
2. Are there certain places/regions in your Territory that bears are associated with?

**2. GRIZZLY BEAR RELATED QUESTIONS:**

**LEK Candidates:**

1. Have you ever seen a grizzly bear on an island?
   1. No response: End interview
   2. Yes response, Ask:
2. How many times have you seen a grizzly bear on an island?
3. Think about the first time you saw a grizzly bear on an island.
   1. What year was it?
   2. Which island was it on? Please draw location on the supplementary map
   3. What season was it?
   4. How many bears did you see?
   5. Could you tell whether they were male or female?
   6. Could you identify the age(s)? Was it a cub, juvenile, or an adult?
4. Since then, what year(s) have you seen a grizzly bear on an island?
5. For each year you saw a grizzly bear on an island please answer the following for each siting event.
   1. What year was it?
   2. Which island was it on? Please draw location on the supplementary map
   3. What season was it?
   4. How many bears did you see?
   5. Could you tell whether they were male or female?
   6. Could you identify the age(s)? Was it a cub, juvenile, or an adult?

**3. Supporting hypotheses of island grizzly bear observations (only for participants who report a grizzly bear on an island)**

1. Since you have seen grizzly bear(s) on islands, have you noticed a change in the number of sightings on the adjacent mainland?
2. Do you think observing grizzly bears on islands is a new phenomenon? If so, do you have any thoughts on what may be causing this apparent change?
3. What changes, if any, took place in the territory before, during and after you noticed (if noted) grizzly bears on islands?

**TEK ONLY:**

1. Through your stories have you ever heard of grizzly bears living on islands?
2. When you were growing up, did your elders ever speak of grizzly bears living on islands?
3. If there was a previous time in which grizzly bears were told to be on islands, do you remember the cause of why they moved there? Were they always there?

**3. PERCEPTIONS OF DISTRIBUTION (*based on all knowledge sources: personal observations, friends/family member sightings, stories, etc*):**

1. **TEK**
   1. Draw an outline of areas you would expect to have seen grizzly bears traditionally (pre-1992) (*ie* if you were asked to draw a map of grizzly bear range for this time period)
2. **TEK and LEK candidates**
   1. Draw an outline of areas you would expect to see grizzly bears during 1992- 2002 (*ie* if you were asked to draw a map of grizzly bear range for this time period)
   2. Draw an outline of areas you would expect to see grizzly bears during 2003- 2012 (*ie* if you were asked to draw a map of grizzly bear range for this time period)

**5. INTERVIEWEE RECOMMENDATION**

i. Do you have any suggestions for other ‘bear experts’ who would be valuable for us to interview?

**6. CLOSING QUESTIONS**

Do you have any additional comments or questions for us?

Also, we want to tell you that you will be able to review draft results and verify your responses. What is the best way to get you this information?

Finally, we want to let you know that the results will be given to your resource management/stewardship office to help inform decision-making.
